# Hints From the Cellular Functions to the Practical Outlook of Circular RNAs

**DOI:** 10.3389/fgene.2021.679446

**Published:** 2021-06-17

**Authors:** Liora Yesharim, Marzieh Mojbafan, Maryam Abiri

**Affiliations:** ^1^Department of Medical Genetics and Molecular Biology, School of Medicine, Iran University of Medical Sciences, Tehran, Iran; ^2^Department of Medical Genetics, Ali-Asghar Children’s Hospital, Tehran, Iran; ^3^Shahid Akbarabadi Clinical Research Development Unit (ShACRDU), Iran University of Medical Sciences, Tehran, Iran

**Keywords:** circRNA, biomarker, biological function, molecular therapy, protein-coding, immunity response, origin of life

## Abstract

Although it has been about 30 years since the discovery of circular RNAs (circRNAs) in mammalian cells, these subtypes of RNAs’ capabilities have come into focus in recent years. The unique structure and various functional roles of circRNAs in many cellular processes have aroused researchers’ interest and raised many questions about whether circRNAs can facilitate the diagnosis and treatment of diseases. To answer these questions, we will illustrate the main known functions and regulatory roles of circRNAs in the cell after presenting a brief history of the discovery of circRNAs and the main proposed theories of the biogenesis of circRNAs. Afterward, the practical application of circRNAs as biomarkers of different pathophysiological conditions will be discussed, mentioning some examples and challenges in this area. We also consider one of the main questions that human beings have always been faced, “the origin of life,” and its possible connection to circRNAs. Finally, focusing on the various capabilities of circRNAs, we discuss their potential therapeutic applications considering the immunity response toward exogenous circRNAs. However, there are still disputes about the exact immune system reaction, which we will discuss in detail.

## Introduction

Our current knowledge of the role and importance of circRNAs in mammals, including humans, has come a long way. Since the first circular form of RNA was found in plant-infected viroids (uncoated RNA molecules that infect certain higher plants), scientist’s views on circRNAs have undergone tremendous changes, thanks to the development of new high-throughput sequencing techniques. Sanger et al. (1976) confirmed that viroids like PSTVd are single-stranded RNA molecules with a circular structure, based on the evidence from electron microscopic observations and biochemical investigation.

Nigro et al. (1991) announced the presence of circular forms of tumor suppressor gene, DCC (deleted in colorectal cancer). They referred to this finding merely as “scrambled exon” (Nigro et al., 1991).

**FIGURE 1:**
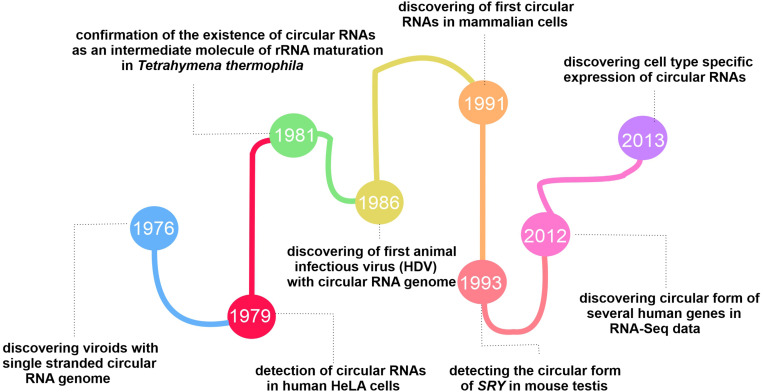
Timeline showing the key events in the history of circRNAs discovery.

The fluctuations in the circRNA discovery area continued until changes in the conventional algorithms and library preparation methods for RNA-seq in 2012, revealing the global expression of circRNAs in the mouse brain and different cell types (Salzman et al., 2012). The tissue-specificity and conserved expression of circRNAs mentioned in two separate published papers in 2013 revealed their functional role in cells (Jeck et al., 2013; Salzman et al., 2013). Since then, researchers have studied circRNAs from different angles to reveal this group of RNAs’ exact function and characteristics (More details on the history behind the evolution of circRNAs researches are presented in Figure 1).

Herein we summarize the known and unknown issues about circRNAs. The possible biogenesis models, roles in different pathologies, and the biological functions of circRNAs have been reviewed earlier (Salzman, 2016; Haddad and Lorenzen, 2019), so we overview these subjects briefly and from different perspectives. This review’s primary focus is on the potential applications of circRNAs, especially in the diagnosis and treatment of diseases, and the findings of studies on the possible immune system response toward the transfection of exogenous circRNAs. Although there are still many unknown issues in circRNAs’s field of research, it is likely that in the near future, given the characteristics of circRNAs and the spread of knowledge in this field of study, a number of the main challenges facing scientists may be addressed, especially in the treatment of diseases.

## RNA Binding Proteins, the Main Contributors of circRNAs Biogenesis

**FIGURE 2:**
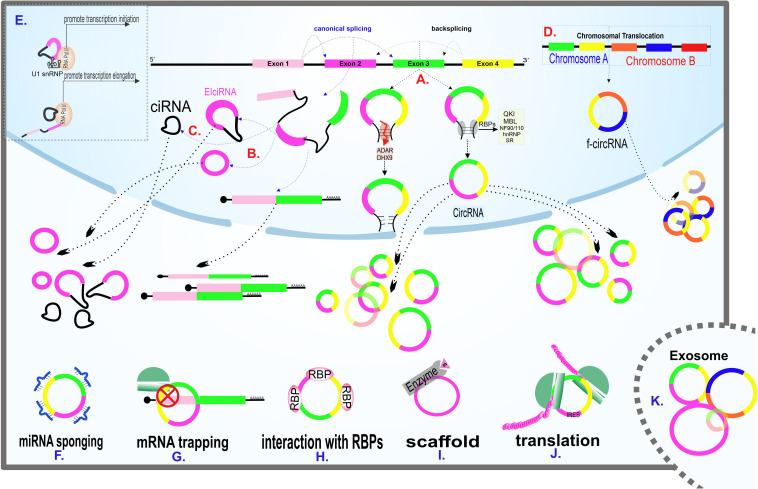
Scheme of main models of circRNAs biogenesis and circRNAs known cellular functions. **(A)** Generation of circRNAs via direct backsplicing and with the help of RBPs. ADAR and DHX9 inhibit circRNAs formation by unwinding RNA base pairings. **(B)** Exon skipping model of circRNAs biogenesis. The intermediate lariat formed in canonical splicing endures backsplicing. **(C)** The remained intron from splicing fails to debranch, leading to CIRNA formation. **(D)** F-circRNA formation following translocation of chromosomes. **(E)** Transcription regulation of circRNAs. EIciRNA interacts with the U1 snRNP subunit of spliceosome machinery at the beginning of transcription. CIRNA interacts with phosphorylated RNA polymerase II, facilitating elongation. **(F)** A circRNA sponging several miRNAs. **(G)** A circRNA traps its linear counterpart and inhibits translation by blocking the translation start site. **(H)** Interaction of a circRNA with RBPs. (**I**) CircRNA acting as a scaffold, facilitating enzyme and substrate reaction. **(J)** CircRNA containing IRES, coding protein. **(K)** Enrichment of various circRNAs in exosomes. Exosomal circRNAs are potential biomarkers for various diseases.

The precise mechanism of circRNAs biogenesis is still a point of discussion. Although spliceosome appears to be the main actor of circRNA biogenesis, this process’s details remained unclear. The two highlighted mechanisms recommended by the researchers are direct backsplicing and exon skipping (lariat model). In the direct backsplicing model, there are inverted repeats in the vicinity of the exons. The base pairing of inverted repeats leads to forming a stem-loop structure, which brings circRNA-forming exons into proximity, thus enhancing the interaction between donor splice-site and upstream acceptor site (instead of downstream acceptor site in canonical splicing) (Figure 2A). Backsplicing seems to be a rare phenomenon due to the lower energy efficiency than canonical splicing and the need for complementary sequences that initiate the backsplicing process by bringing the splice sites close to each other (Zhang et al., 2016). Emerging shreds of evidence suggest that the complementary sequence is not enough for circularization, and backsplicing is assisted by many RNA binding proteins (RBPs).

One of the most prominent RBPs contributing to the biogenesis of circRNAs is the splicing factor, namely Quaking (QKI). QKI, which is a member of the STAR family (a gene family involved in signal transduction and activation of RNA), binds to conserved binding sites in the introns adjacent to the circularization location and seems to catalysis the circularization process by dimer formation (Vernet and Artzt, 1997; Conn et al., 2015).

Muscleblind (MBL) -examined in Drosophila- is another splicing factor involved in circRNA’s biogenesis. MBL acts in a cis manner and binds to its conserved binding sites, and promotes the synthesis of circMbl, the most abundant circRNA in the Drosophila’s head. The feedback loop occurring in this process is remarkable. The MBL protein -synthesized from a linear Mbl transcript- helps create circular Mbl and prevents linear counterpart formation by competition with the canonical splicing (Ashwal-Fluss et al., 2014).

NF90/NF110, double-strand binding proteins originating from *the ILF3* gene, also contribute to the backsplicing process. Studies indicated that circRNA’s overall expression decreases during viral infection, partly due to these antiviral operators’ nuclear export. Experiments such as the biotin-labeled RNA pull-down assays have shown that these proteins strongly bind to dsRNA and probably facilitate circRNA biogenesis by stabilizing RNA base pairing (Li et al., 2017).

Other facilitators of the backsplicing process include the two protein families called heterogeneous nuclear ribonucleoprotein (hnRNPs) and serine-arginine (SR). Previous studies have examined the role of hnRNPs in processes such as pre-mRNA maturation, cellular transport, and stabilizing of mRNA (Geuens et al., 2016). SR proteins also have multiple similar roles in regulating gene expression (Shepard and Hertel, 2009). SR and hnRNPs act together to circularize genes like *Drosophila laccase2* (Kramer et al., 2015).

However, RBPs do not always accelerate the backsplicing process and circularization. Both ADAR (adenosine deaminases acting on RNA) and an RNA helicase called DHX9 can interact with inverted *ALU*s repeats and unwind RNA base pairing and, as a result, inhibit circularization (Ivanov et al., 2015; Aktaş et al., 2017).

As mentioned earlier, backsplicing is not the only possible mechanism of circRNA biogenesis. In the exon skipping (lariat) model, following canonical splicing, the intermediate lariat includes skipping exons endured backsplicing (Figure 2B). In this potential biogenesis model, repeated sequences surrounding the exons are unnecessary (Zaphiropoulos, 1997; Barrett et al., 2015).

Two other probable mechanisms of circRNA’s biogenesis are failure to debranch intron lariat (Figure 2C) and translocation (Figure 2D). Translocations mainly occur in cancers and are believed to be the potential biogenesis mechanisms (Guarnerio et al., 2016; Wilusz, 2017). In addition to the items mentioned earlier, the RNA pol II speed of synthesis also plays a decisive role in a transcript’s fate. The higher the RNA pol II elongation rate, the higher the ratio of circRNA to linear RNA synthesis. As the percentage of RNA synthesis increases, the pairing of complementary sequences is facilitated (Zhang et al., 2016).

## circRNAs Play Fundamental Roles in Cell Operation

Discovering facts such as circRNAs tissue-specific expression and specific expression patterns during various development stages has led researchers to investigate the functions of circRNAs.

Herein, we will give a brief overview of some of the main functions that have been described for circRNAs.

### miRNA Sponging, Important but Not General

MicroRNAs (miRNAs), RNA molecules with an approximate length of 22 nucleotides, are involved in post transcription regulation of genes by preventing their target mRNAs’ translation (Ambros, 2004). However, the regulatory network across the transcriptome does not end up with mRNAs and miRNAs. In 2013, an investigation showed an ability of circRNAs in miRNA sequestering, the phenomenon known as “miRNA sponging” (Hansen et al., 2013; Figure 2F). The miRNA sponge capability is not limited to circRNAs, and this feature is because of the presence of MREs (MiRNA Response Elements). CircRNAs are also referred to as competitive endogenous RNAs (ceRNAs) in some published data through their ability to inhibit miRNAs’ regulatory function on mRNAs (Mitra et al., 2018; Smillie et al., 2018). Although a significant number of published papers in the field of circRNA rely on this possible role, this phenomenon has been confirmed in just a small number of circRNAs (Dori and Bicciato, 2019). CiRS-7 (also known as CDR1as), circSRY, and circHIPK3 are three circRNAs involved in sponging miR-7, miR-138, and miR-124, respectively (Hansen et al., 2013; Zheng et al., 2016). CircRNAs indirectly affect extensive gene networks. Each circRNA could have several miRNA bindings sites, even for multiple different miRNAs, and each miRNA could inhibit numerous mRNAs. To attribute the sponging role to circRNAs, there must be evidence of miRNA-circRNA co-localization. Additionally, co-expression of circRNA and the related miRNA and the presence of MREs is necessary. Furthermore, the circRNA’s length and the adequacy of their expression abundance should be considered (Hansen et al., 2013; Dori and Bicciato, 2019).

### Circular RNAs Regulate Gene Expression in Various Ways

CircRNAs are involved in several gene expression regulation stages, such as transcription initiation and elongation, splicing, and even translation. The three major known subtypes of the circRNAs, exonic circRNAs (EcircRNA), intronic circRNAs (CIRNA), and exon-intron circRNAs (EIciRNA), play roles in this area.

The existence of 2’–5’ Phosphodiester-linked in CIRNAs supports the hypothesis that the formation of this particular subgroup of circRNAs happened because of lariat debranching failure (Figure 2C). CIRNAs are enriched in the nucleus and positively interact with phosphorylated RNA pol II during elongation (Zhang et al., 2013; Figure 2E).

EIciRNAs, which contain both intron and exon in their structure, are also accumulated in the nucleus and indirectly regulate the RNA pol II by RNA-RNA interaction with the U1 snRNA subunit of spliceosome machinery and aid initiation of transcription (Li Z. et al., 2015; Figure 2E). Both CIRNAs and EIciRNAs apply for their regulatory role in a cis manner and enhance their parental gene transcription. The trans-regulatory role of these circRNAs is still a matter of debate.

CircRNAs are also involved in post-transcription regulation. Except for the sponge role of circRNAs described earlier, circular transcripts of a gene can prevent their linear counterparts’ efficient expression in other main ways. One way is the competition between the linear and the circular form of a gene in binding to translation-facilitating proteins. CircPABPN1, for example, appears to reduce the binding of HuR protein -which is known as translation promoter of PABPN1 mRNA- to its linear counterpart and thus reduces the efficiency of translation (Abdelmohsen et al., 2017). There is also a theory in which CircRNAs can prevent protein buildup in a phenomenon called “mRNA trapping.” CircRNAs like circFMN trap their linear mRNA counterparts and block the translation start site, thus protein synthesis (Chao et al., 1998; Figure 2G). Also, given that both linear and circular forms of a gene originating from the same splice site, the biogenesis of circRNAs decreases the final expression rate of linear RNA. It has been found that synthetic cirRNAs are not only transmissible to the cytoplasm but also the nucleus. Given that circRNAs are capable of regulating processes in the nucleus, it is possible to use circRNAs to regulate processes such as transcription and splicing (Jost et al., 2018).

### Cell Directors in Close Contact With circRNAs

CircRNAs interact with proteins, the important cellular directors, in a variety of ways (Figures 2H,I). In addition to the above-mentioned RBPs-circRNAs interactions during circRNAs biogenesis, circRNAs can act as a protein transporter, subcellular localizer and scaffold, facilitating protein-protein interactions.

Although bioinformatic studies were done based on nucleotide sequences, the RBP chances of binding to circRNAs are no more than linear forms, but the interaction seems stronger (You et al., 2015).

Transfected cells with circAmotl1 showed increased AKT levels in the nucleus, supporting the fact that circAmotl1 may be involved in the nuclear translocation of AKT protein (Zeng et al., 2017). Previous research has investigated the potential interaction between circRNAs and proteins, denoted that circ-Foxo3 can potentially act as a scaffold and facilitate the interaction of CDK2 and its inhibitor, p21. The ternary complex formed of circ-Foxo3, p21, and CDK2, blocked the G1 to S phase transition of the cell cycle by inhibiting the CDK2 and cyclin A/cyclin E interactions (Du et al., 2016).

Evaluation of the role of circANRIL in atherosclerosis and ribosomal RNA (rRNA) maturation process using mass spectrometric and RNA immunoprecipitation (RIP) assays showed two proteins; PES1 and NOP14 interacted mostly with circANRIL, respectively (Holdt et al., 2016). Since the interaction between PES1 and rRNA is essential for rRNA’s maturation and ribosome formation, circANRIL binding to this member of the PeBoW complex blocks the rRNA binding site and consequently impair protein translation rate and cell growth (Holdt et al., 2016).

Cells perform their vital functions through proteins. Proteins interact with circRNAs for a variety of purposes. As can be deduced from the above examples, some of the most important proteins are transported in the cell with the help of circRNAs. Besides, proteins can interact effectively with other proteins and molecules by binding to circRNAs as a platform. That is one of the many reasons why the cell faces a crisis if mutations affect the expression levels or RNA binding sites of circRNAs.

### CircRNAs, Blurring Boundary Between Coding and Non-coding World

CircRNAs are classified in the non-coding RNAs category. However, evidence from mass spectrometry, ribosomal footprinting, and CRISPR Cas9 based experiments indicated this subtype of RNAs’ potential to produce proteins (Legnini et al., 2017; Pamudurti et al., 2017; Yang Y.Y. et al., 2017). Two general mechanisms are known to initiate protein synthesis: cap-dependent and cap-independent [IRES (Internal Ribosome Entry Site)-mediated] translation.

Since there is no 5′ cap structure in circRNAs, the probable mechanism for circRNA translation is the cap-independent (Chen and Sarnow, 1995), and it is done with the help of a specific RNA element called IRES, which enables the interaction of the 40S ribosomal subunits to the transcript (Merrick, 2004; Figure 2J). The cap-independent translation is less effective than the cap-dependent mechanism, but is more commonly used during cellular stress like hypoxia and nutrient starvation, which are common in cancer cells (Walters and Thompson, 2016). The cap-independent mechanism promotes the translation of proteins, such as transcription and growth factors, required for cell survival (Walters and Thompson, 2016; Diallo et al., 2019; Zhao et al., 2019). Studies have shown that sequences containing methylated adenosine N6 (m6A) site at 5’ UTR can act as IRES (Yang Y. et al., 2017).

M6A modifications involvement in processes such as mRNA stability, splicing, and translation were approved, and now it seems that m6A is also effective at the beginning of circRNAs translation (Wang et al., 2014; Ma et al., 2016; Yang Y. et al., 2017; Zhou et al., 2019). CircRNA-m6A-seq revealed that m6A motifs are enriched in circRNAs. Yang Y. et al. (2017) showed that circRNAs containing even a single m6A motif are efficiently transcribed. Inserting m6a motifs before the start codon of circRNA reporter results in efficient GFP (green fluorescent protein) translation. The research revealed that the production of proteins from circRNAs containing m6A modification, which increased by heat shock, is similar to the translation of mRNAs containing m6A modifications. This study also demonstrated that the YTHDF3 plays an indispensable role in this process as an m6A reader, which interacts with the translation initiation factor eIF4G2 (Yang Y. et al., 2017).

Several studies have shown the potential role of circRNAs in protein production (Legnini et al., 2017; Zhang et al., 2018; Liang et al., 2019). Also, tools have been developed to predict the protein-coding potential of circRNAs. For example, CircCode is a python-based tool capable of predicting protein production in humans and *Arabidopsis thaliana* (Sun and Li, 2019).

It seems that by the advancement of technology in various fields of science and the elimination of technical problems in this area of research, more substantial evidence may be found to prove the ability of circRNAs to code protein.

As can be seen from the examples mentioned earlier, circRNAs are involved in different levels of cell metabolism. Understanding these functions and their effect on maintaining cell homeostasis can inspire new therapies. For example, targeting a circRNA, which is multidimensionally involved in the pathogenesis of a disease, can help effectively treat that disease. Even more, designing and transfecting a circRNA that is capable of adequately coding a tumor suppressor protein and also targeting multiple onco-miRs can be a valuable treatment method.

## circRNA’s unique features nominated them as the origin of life

Features like development and tissue specificity, self-catalyzing capacity, and being conserve are among different species are mentioned for circRNAs. However, perhaps the most essential feature is the property acquired through their specific structure (Xia et al., 2017). The closed ring structure of circRNAs leads to the absence of a polyadenylated tail and 5’–3’ polarity. As a result, they are highly stable because of their resistance to exonucleases, including RNaseR (Suzuki et al., 2006). On average, the half-life of circRNAs is 2.5 times more than their linear counterpart, and this stability can last up to 48 h in cells (Jeck et al., 2013; Enuka et al., 2015). Considering this capability, circRNAs could answer the old question “where and how life begins?.” RNA molecules were introduced as a possible origin of life because they could have catalytic activity and store genetic information. However, one critique of the “RNA World Hypothesis” is that RNAs are not stable enough. Given the roles and properties mentioned for circRNAs, especially the super stability feature of these molecules, they can be considered as the potential candidate molecules in initiating life (Demongeot and Moreira, 2007; Neveu et al., 2013). However, answering such a comprehensive question is very challenging since there is no direct evidence of this process. Ideally, the primary circRNA should have a minimum number of nucleotides to minimize the possibility of breakage. It must also be capable of forming secondary structures such as hairpins to increase both the molecule’s stability and have the surface for interaction (Demongeot and Moreira, 2007). Putting aside questions about how early nucleotides formed on Earth and how nucleotides bind to each other in the “prebiotic pool,” this hypothesis seems to be worth further investigation.

## CircRNAs, Potential Biomarkers, and the Challenges Ahead

CircRNAs have been suggested as potential biomarkers, as their role in several intracellular molecular processes becomes apparent. Exceptional stability among all types of RNAs is perhaps the most important reason for using circRNAs as biomarkers. CircRNAs, especially those originated from exosomes (Figure 2K), can be detected in various body fluids, including saliva and serum, making it possible to diagnose and predict the prognosis of different kinds of diseases in a non-invasive or minimal-invasive manner (Li Y. et al., 2015). Some circRNAs suggested as the biomarker in various cancers and non-cancerous diseases with reported specificity and sensitivity are listed in Tables 1, 2.

**TABLE 1:** Potential circRNA biomarkers in different types of cancers.

CircRNA ID	Alias	Cancer type	Sensitivity	Specificity	Biological sample	Regulation	Clinicopathologic correlation	References
hsa_circ_0004585	hsa_circRNA_004585	Colorectal cancer	85.1% 90.8%	51.1 40.8%	Tissue Plasma	Up	Tumor size	Tian et al., 2019
hsa_circ_0006988	hsa_circRNA_100799	Pancreatic cancer	57.38%	70.49%	Tissue and plasma	Up	Venous invasion, lymphatic invasion	Yang F. et al., 2017
hsa_circ_103110 hsa_circ_006054 hsa_circ_100219	− hsa_circRNA_060540 −	Breast cancer	63.0% 65.0% 69.0%	63.0% 69.0% 71.0%	Tissue	Up Down Down	Venus invasion, Metastasis	Lü et al., 2017
hsa_circ_0000190	hsa_circRNA_000190	Gastric cancer	72.1%	68.3%	Tissue and plasma	Down	Tumor diameter, Lymphatic metastasis, Distal metastasis	Chen S. et al., 2017
hsa_circ_0067582	hsa_circRNA_103482	Gastric cancer	66.6%	61.2%	Tissue	Down	Tumor diameter	Yu et al., 2019
hsa_circ_0065149	hsa_circRNA_103342	Gastric cancer	79.2%	61.5%	Tissue	Down	Larger tumor diameter, Perineural invasion	Shao et al., 2019
hsa_circ_0005556	hsa_circRNA_102631	Gastric cancer	64.0%	82.0%	Tissue	Down	Differentiation, TNM stage, Lymphatic metastasis	Yang et al., 2019
hsa_circ_002059	hsa_circRNA_020596	Gastric cancer	81.0%	62.0%	Tissue	Down	TNM stage, Distal metastasis	Li P. et al., 2015
hsa_circ_0000467	hsa_circRNA_101231	Gastric cancer	70.5%	64.8%	Tissue	Up	Lymphatic invasion, TNM stage	Lu et al., 2019
hsa_circ_0000567	hsa_circRNA_101436	Colorectal cancer	83.3%	76.4%	Tissue	Down	Tumor size, Lymph metastasis, Distal metastasis, Tumor node metastasis, TNM stage	Wang et al., 2018
hsa_circ_0005962 hsa_circ_0086414	hsa_circRNA_104667 hsa_circRNA_104736	Lung adenocarcinoma	71.9% 77.1%	72.2% 66.6%	Plasma	Up Down	Proliferation	Liu X.X. et al., 2019
hsa_circ_0013958	hsa_circRNA_100323	Lung adenocarcinoma	75.5%	79.6%	Tissue	Up	TNM stage, Lymphatic metastasis	Zhu et al., 2017
hsa_circ_0137287	−	Papillary thyroid carcinoma	79.2%	90.0%	Tissue	Down	Extrathyroidal extension, Lymph node metastasis, T stage, Larger tumor diameter	Lan et al., 2018
hsa_circ_0003998	hsa_circRNA_003998	Hepatocellular carcinoma	84.0%	80.0%	Tissue	Up	AFP level, Larger tumor diameter, Microvascular invasion, Differentiation	Qiao et al., 2019
hsa_circ_0004018	hsa_circRNA_101940	Hepatocellular carcinoma	71.6%	81.5%	Tissue	Down	AFP level, Tumor diameters, Differentiation, Tumor-node-metastasis, TNM stage	Fu et al., 2017
hsa_circ_0128298	−	Hepatocellular carcinoma	67.4%	80.5%	Tissue	Up	Vascular cancer embolus, Lymphatic metastasis, Organ metastasis	Chen et al., 2018
Hsa_circ_0001649	hsa_circRNA_104206	Hepatocellular carcinoma	81.0%	69.0%	Tissue	Down	Tumor size, Tumor embolus, Metastasis	Qin et al., 2016
hsa_circ_0072387	hsa_circRNA_103829	Oral squamous cell carcinoma	71.4%	69.8%	Tissue	Down	Tumor diameters, T stage, TNM stage	Dou et al., 2019
hsa_circ_0001946	hsa_circRNA_105055	Esophageal squamous Cell cancer	92.0%	80.0%	Plasma	Down	Proliferation, Migration, Invasion	Fan et al., 2019

**TABLE 2:** Potential circRNA biomarkers in non-cancerous diseases.

CircRNA ID^a^	Alias	Disease	Sensitivity	Specificity	Biological sample	Regulation	Probable mechanism of action^b^	References
hsa_circ_0007121	−	Preeclampsia	77.3%	70.3%	Plasma	Down	Act in apoptosis, Wnt-signaling, and HIF-1 pathways	Bai et al., 2018
hsa_circ_0000479	hsa_circRNA_000479	Systemic lupus erythematosus (SLE)	80.0%	71.1%	Peripheral blood mononuclear cells	Up	Not included	Luo et al., 2020
hsa_circ_0044235	hsa_circRNA_044235	Systemic lupus erythematosus (SLE)	70.0%	100%	Peripheral blood mononuclear cells	Up	possible interaction with hsa-miR-892a	Luo et al., 2019b
hsa_circ_0000086 hsa_circ_0076767	hsa_circRNA_001264 hsa_circRNA_104121	Primary Sjögren’s syndrome	80.0% 73.3%	73.3% 70.0%	Peripheral blood mononuclear cells	Up	possible interaction with miR-18a-3p possible interaction miR-203a-3p and miR-143-3p	Su et al., 2019
hsa_circ_0063411	hsa_circRNA_063411	Amyotrophic Lateral Sclerosis (ALS)	100%	100%	Plasma	Up	possible interaction with hsa-miR-647	Dolinar et al., 2019
hsa_circ_0000700	hsa_circRNA_001937	Active Tuberculosis	85.0%	77.5%	Peripheral blood mononuclear cells	Up	possible interaction with 6 miRNAs (includes miR-22-5p, miR-26b-3p, miR-10b-3p, miR-376a-5p and miR-597-3p) MiR-26b act in the inflammatory response by modulating the NFκB pathway through targeting PTEN.	Huang et al., 2018
hsa_circ_0002453	circRNA_002453	Lupus nephritis	90.0%	84.1%	Plasma	Up	Not included	Ouyang et al., 2018
hsa_circ_0126991	−	Essential Hypertension	72.4%	67.3%	Plasma	Up	Act in cell apoptosis and the Robo receptor signaling pathway and related to intercellular adhesion factors.	Liu L. et al., 2019
Hsa_circ_0035197 Hsa_circ_0002715	hsa_circRNA_101515 hsa_circRNA_103150	New-Onset Rheumatoid Arthritis	71.2% 57.6%	68.6% 77.1%	Peripheral blood mononuclear cells	Up	possible interaction with hsa-miR-378d and hsa-miR-26b-3p	Luo et al., 2019a
hsa_circ_0054633	−	Type 2 Diabetes Mellitus (T2DM)	55.0%	85.0%	Peripheral blood mononuclear cells	Up	Possible role in the biological processes like cell cycle and mitotic cell cycle arrest and catabolism of molecules.	Li et al., 2016
hsa_circ_0068481	hsa_circRNA_103544	Pulmonary arterial hypertension	74.39%	98.7%	Serum	UP	Not included	Zhang et al., 2019
hsa_circ_0084021	hsa_circRNA_104597	Schizophrenia	84.31%	86.4%	Peripheral blood mononuclear cells	Down	Possible interaction with hsa-miR-659-3p, hsa-miR-548d-5p, hsa-miR-651-3p, hsa-miR-548c-5p and hsa-miR-548a-5p.	Yao et al., 2019
hsa_circ_0032131	−	Osteoarthritis	90.0%	65.0%	Peripheral blood mononuclear cells	Up	Possible interaction with 50 miRNAs	Wang Y. et al., 2019
hsa_circ_0001879 hsa_circ_0004104	hsa_circRNA_103987	Coronary artery disease	83.1% 70.7%	54.3% 61.4%	Peripheral blood mononuclear cells	Up	Possible role in metabolic pathways and PI3K-Akt signaling pathways.	Wang L. et al., 2019
hsa_circ_0124644	−	Coronary artery disease	86.7%	76.7%	Peripheral blood mononuclear cells	Up	Possible interaction with miR-10a-5p	Zhao et al., 2017

**^*a*^CircRNA ID in circBase database. ^*b*^Possible mechanism of action indicated by bioinformatic analysis.**

RNA-seq and microarray are the two common high throughput methods for analyzing RNA expression profiles. Using the RNA-seq method seems to be more challenging and less efficient than microarray in the field of circRNAs. Using different library preparation methods, such as size selection or the RNA-seq reading depth, can lead to the loss of small-sized and low-abundant circRNAs data, respectively. Besides, although various algorithms have been developed for circRNA detection in RNA-seq data, none of them seems to be as efficient as they should be, and there is a slight overlap between the results of different algorithms (more details on Szabo and Salzman, 2016). It was demonstrated that microarrays are about 10 times more capable of detecting circRNAs than RNA-seq with an average of 20 million reads depth. However, it is impossible to detect unknown circRNAs using microarrays (Li et al., 2018).

Another factor making the study of circRNAs demanding is the lack of a unit and standard nomenclature system (Franz et al., 2018). Various databases and articles use different naming methods, and converting names to each other is time-consuming and complicated; therefore, there is an urgent need for a single nomenclature system. Some of the most widely used databases for circRNAs and the information they provide is listed in Table 3.

**TABLE 3:** Some of the most useful circRNA databases which are constantly updated.

Database/webtool	Website	Data provided and main capabilities	Covered species	References
circBase	http://www.circbase.org/	Unique circRNA ID, position and genomic range of circRNAs, can be searched by three different ways: (1) Simple search, (2) List search and (3) Table browser	*Homo sapiens, Mus musculus, Caenorhabditis elegans, Latimeria*	Glažar et al., 2014
circRNADb	http://reprod.njmu.edu.cn/circrnadb/	CircRNAs with protein coding potential by considering ORF and IRES elements, can also be browsed by 11 different cell type and tissue. General information like genomic position, best transcript id and etc. provided	*Homo sapiens*	Chen et al., 2016
TSCD (Tissue-Specific CircRNA Database)	http://gb.whu.edu.cn/TSCD	Validated tissue specific circRNAs potential related miRNAs and proteins considering MREs and protein binding sites, conservation among species	*Homo sapiens, Mus musculus*	Xia et al., 2017
CSCD (Cancer-Specific CircRNA Database)	http://gb.whu.edu.cn/CSCD	Cancer specific circRNAs Function prediction considering protein coding capacity and miR sponging potential cellular location	*Homo sapiens*	Xia et al., 2018
CIRCpedia v2	https://www.picb. ac.cn/rnomics/circpedia/	Visualization of circRNAs circRNA Conservation among Homo sapiens and Mus musculus general circRNAs information	*Homo sapiens*, *Mus musculus*, *Rattus norvegicus*, *Danio rerio*, *Drosophila melanogaster, Caenorhabditis elegans*	Dong et al., 2018
CircR2Disease	http://bioinformatics.zju.edu.cn/Circ2Disease	Curated disease-related circRNAs Function description (validated or predicted miR or RBPs interaction) Expression pattern	*Homo sapiens*	Fan et al., 2018
TRCirc	http://www.licpathway.net/TRCirc/	Information on transcription regulatory function of circRNAs based on ChIP-seq data visualization of TF–circRNA regulation network and Histogram of expression of circRNAs are possible	*Homo sapiens*	Tang et al., 2019
WebCircRNA	https://rth.dk/resources/webcircrna/	Predict the circRNA production possibility based on sequence and conservation	All species	Pan et al., 2018
CircInteractome	https://circinteractome.nia.nih.gov/	circRNA-miR and circRNA_RBP predicting tool Primer and siRNA design capability	*Homo sapiens*	Panda et al., 2018
CircFunBase	http://bis.zju.edu.cn/CircFunBase/	Collection of functional circRNAs Sequence blast available	15 model species Including *Homo sapiens, Macaca mulatta and Arabidopsis thaliana*	Meng et al., 2019
Circbank	http://www.circbank.cn/	Use the naming system for circRNAs based on the host gene Predict miR sponging and protein coding capability, m6A methylation, conservation across species	*Homo sapiens*	Liu M. et al., 2019
circAtlas	http://circatlas.biols.ac.cn/	Conservation information of circRNAs across the species, tissues and individuals Functional annotation of the circRNAs based on miRNA interaction and RNA-binding protein (RBP) interaction data. The database can be searched in different ways like genomic position, ensemble gene ID and different circRNA names.	Si$x vertebrates including *Homo sapiens, Macaca mulatta, Mus musculus, Rattus norvegicus, sus scrofa, Gallus gallus*	Wu et al., 2020

## Therapeutic Utility of CircRNAs

**FIGURE 3:**
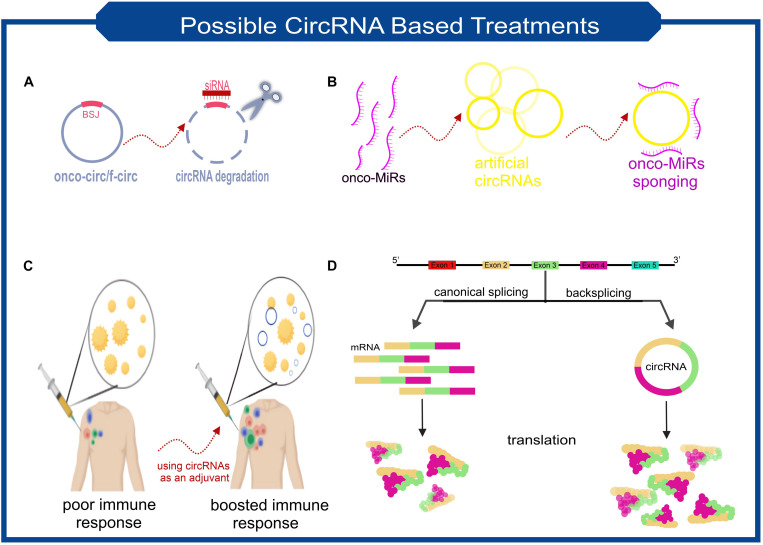
Scheme of some the possible treatment approaches based on the characteristics of circRNAs. **(A)** Targeting the backsplice junction of onco-circs or f-circs by siRNAs leads to the degradation of these harmful circRNAs. **(B)** Exclusive circRNAs designed to sequester/sponge oncogenic miRNAs. **(C)** Utilizing the possible immunogenic properties of circRNAs as an adjuvant. **(D)** Despite the lower biogenesis of circRNAs than linear RNAs, more stable translation can be achieved due to the high stability of circRNAs. This feature makes circRNAs ideal for protein replacement therapies.

CircRNAs are molecules with the potential to treat a wide range of diseases. CircRNA-based treatments can be divided into two general categories. Not only can they be used as a therapeutic target, but it is also possible to take advantage of engineered circRNAs as medicinal operators (Figure 3 summarized some potential applications of circRNAs in the treatment of diseases).

Imagine a circRNA that is known to play a significant role in the exacerbation of a disease. One therapeutic approach can be to knockdown this circRNA with the help of RNA interference (RNAi) (Figure 3A). siRNA’s must be designed to target back-spliced junction; thus, the linear counterpart remains intact (Santer et al., 2019). Studies confirmed that using siRNAs-based knockdown of circRNAs involving various diseases’ pathogenesis will be a promising approach. *In vitro* assays demonstrated that circ-EIF4G3 and circ-0023642 knockdown would suppress cell proliferation and invasion of gastric cancer (Zhou et al., 2018; Wang Q. et al., 2019). Similar experiments in knockdown of circ-VAPA and circ_ANF609 have similar colorectal and nasopharyngeal carcinoma results, respectively (Yu, 2020; Zhang et al., 2020).

Fusion circRNAs (f-circRNAs), primarily generated in chromosomal translocations during tumorigenesis, are potential cancer treatment targets. The formation of circRNAs can lead to drug resistance, cellular transformation, and other tumor-related hallmarks (Guarnerio et al., 2016). F-circRNAs knockdown would be a good choice for cancer cell-specific targeting (Figure 3A). Sometimes circRNAs knockdown can be used as a co-medication. A recently published study found that circRACGAP1 suppression could be used to reduce the toxic effects of apatinib (a drug that has anti-cancer effects by inhibiting VEGFR-2 tyrosine kinase). CircRACGAP1 enables autophagy-related gene 7 (*ATG7*) overexpression by sponging miR-3657. Inhibition of circRACGAP1 leads to cell apoptosis instead of autophagy, which sometimes causes cancer cells to survive under environmental stress (Yun and Lee, 2018; Ma et al., 2020).

Another role of circRNAs, which make them suitable candidates for disease treatment, lies in their ability to act as miRNA sponges (Figure 3B). A recently *in vivo* research result demonstrated that a small dose of unmodified circRNA could sponge two miRNAs involved in cardiac hypertrophy, thereby preventing heart failure (Lavenniah et al., 2020). It has also been shown that transfection of synthetic circRNA named scRNA21 increases the level of miR-21 target proteins such as Daxx by efficiently sponging miR-21 and inhibiting the proliferation of gastric cancer cells (Liu et al., 2018).

Perhaps the most revolutionary circRNA-based treatment lies in their stability and protein-coding capacity. Obtaining a stable expression of an exogenous protein is one of the main issues scientists have been facing. However, using mRNAs for this purpose has the advantage of being safer than DNA manipulation, but achieving adequate and stable expression has always been a challenge for researchers. Considering the potential ability of circRNAs to be translated and their high stability makes them applicable candidates. Researchers found an exogenous circRNA could produce much higher amounts of protein than unmodified linear mRNA and even modified linear mRNA counterparts (Figure 3D; Wesselhoeft et al., 2018). Unmodified circRNAs made from cloned self-splicing intron were transfected using a cationic lipid reagent. The protein production half-life of the highly purified circRNA was approximately two times higher than linear mRNA (Wesselhoeft et al., 2018).

## Immune Response Toward Exogenous circRNAs

The ability of circRNAs in the prolonged production of proteins highly suites them as protein expression vectors and favorable in treating many diseases. Nevertheless, there are still fundamental questions to be answered. How does the immune system respond to exogenous circRNAs? Do circRNAs trigger the immune system as much as linear RNAs?

The truth is that researcher’s findings seem contradictory. A study done before investigating the ability of engineered circRNAs in protein production regarded exogenous circRNAs as an immunogenic agent. Chen Y.G. et al. (2017) showed that transfection of HeLa cells with RNAse R purified circRNAs results in overexpression of innate immunity genes like RIG-I (retinoic-acid-inducible gene-I) up to 500-fold. The role of RIG-I in controlling viral infections had been identified earlier. RIG-I triggers the innate immune system after recognizing dsRNA and 5′ triphosphate (Kell and Gale, 2015). CircRNAs lack 5′ triphosphate, so what triggers innate immunity, and how RIG-I sense endogenous circRNAs from exogenous ones? (Chen Y.G. et al., 2017) proved by an experiment that splicing of a circRNA with human introns in human cells does not induce RIG-I, and this result is independent of circRNA exon sequence. These results stated that RBPs associated with human-specific splicesome mark circRNAs as “self” or “non-self.” Nevertheless, two separate studies, both conducted by Wesselhoeft et al. (2018, 2019), assumed that the innate immune system provocation was only because of circRNAs contamination with other types of RNAs, and the use of ultra-pure circRNAs showed no significant sign of innate immune system provocation. Injection of RNaseR and phosphatase treated circRNAs, purified by HPLC into mice adipose tissue, did not show cytokine secretion. Also, the protein expression remained stable, supporting the ability of circRNAs to perform protein replacement therapy (Ma et al., 2016).

It is not the end of the story. In another research, Chen et al. (2019) confirmed their findings of circRNAs immunizing capacity. In this study, even though using HPLC purified circRNAs, immune system stimulation was still observed. Subcutaneous injection of exogenous circRNA results in dendritic cell activation and, as a result, indirect activation of T cells, which means not only the innate immune system but also the acquired immunity is triggered by exogenous circRNAs. These findings introduce another therapeutic potential of circRNAs to act as an adjuvant (Figure 3C). They also described the role of m6A modification patterns in distinguishing between endogenous and exogenous circRNAs. M6A modifications prevent RIG-I binding to circRNAs and, as a result, activation of the immune response. Experiments indicate that circRNAs from different origins (i.e., human vs. phage) have different m6A modification patterns at 3′ of back-splice junction and, therefore, different immunity response.

Differences in these researches findings may be attributed to details in the experiment’s design, such as cell types or the used reagents (Basavappa and Cherry, 2019). Further studies are needed to confirm each of these results and explain the underlying mechanisms of differences.

## Discussion

For years, circRNAs were known as a “by-product of splicing” rather than a subspecies of RNAs with independent roles. However, in recent years series of functions have been defined for them.

This review summarized the current and potential applications of circRNAs based on their various functions and features. CircRNAs have multiple roles in biological processes, like gene expression regulation, interacting with proteins for different purposes, and coding proteins. Most of the published papers focused on the post transcription regulation of circRNAs, miR sponging. However, researchers should consider items such as sufficient circRNA amount, co-expression with the target miR, and the ratio between circRNA and target miRs in cases where the “sponging” capacity has not been investigated by experimental methods (Dori and Bicciato, 2019). In addition to the fact that circRNAs have various roles in the pathogenesis of various diseases, the stability and presence of circRNAs in body fluids like plasma and saliva have made them ideal diagnostic/prognostic biomarkers, and even several clinical trials are underway in this regard (ClinicalTrials.gov number: NCT04584996; NCT03170830). However, there are still challenges that need to be addressed to expedite and facilitate such studies. RNA-seq and microarray are among the high-throughput technologies that are used to screen differentially expressed circRNAs in different conditions. RNA-seq can evaluate the expression levels of unknown circRNAs. However, different analysis algorithms and even different library preparation methods lead to different results. To get the most out of this method, developing a gold standard for library preparation and bioinformatics analysis pipelines is highly recommended (Engström et al., 2013; Szabo and Salzman, 2016). Although microarray seems to be more efficient than RNA-seq, however, this method is limited to known circRNAs, and also more initial amount of RNA is needed (Li et al., 2018).

In addition to the role that circRNAs can play in the diagnosis and management of diseases, various features of circRNAs can also be used in the treatment of diseases. One possible treatment approach is to reduce the expression of circRNAs such as f-circRNAs and onco-circRNAs using siRNA designed for backspliced junction (Guarnerio et al., 2016). The alternative method for suppressing the circRNA expression is the use of CRISPR/CAS9. Adequate care must be taken in the use of the CRISPR/CAS9 system. Given that most circRNAs have a common origin with their linear counterpart, this method can only be used to remove the complementary sequences required for the biogenesis of circRNAs (Zhang et al., 2016). Moreover, engineered circRNAs can be used to sponge the disease-related miRNAs. Although anti-miRNA drugs such as Miravirsen (a locked-nucleic-acid (LNA)/DNA-mixmer oligonucleotide use to target HCV-related miRNA, miR22) have already been designed, LNA-based drugs show side effects related to the accumulation of unmetabolizable LNA nucleotides. Since the elimination cycle of circRNAs is naturally embedded in the cells, such complications are unlikely to occur (Janssen et al., 2013; Jost et al., 2018).

Another potential revolutionary circRNA-based treatment is achieving stable and efficient protein production in cells, regarding the resistance of circRNAs toward many ribonucleases. Nevertheless, further research needs to be done to establish the immune system’s precise mechanism of action toward exogenous circRNAs. If the immune system is not stimulated, stable protein expression is achieved. But if the activation of the immune system occurs, as happened in Chen et al. (2019) investigation, this event can be used positively, and circRNAs can be used as an adjuvant.

It is possible that by eliminating technical shortcomings and expanding knowledge in this area, circRNAs, once considered a by-product of splicing, shed new light on diagnosing and treating diseases.

## Author Contributions

LY performed the literature search and drafted the manuscript. MA and MM critically revised the work. All authors read and approved the final manuscript.

## Conflict of Interest

The authors declare that the research was conducted in the absence of any commercial or financial relationships that could be construed as a potential conflict of interest.
